# Primary cystic and solid neuroendocrine tumor of the retroperitoneum: A case report

**DOI:** 10.1097/MD.0000000000024054

**Published:** 2021-01-15

**Authors:** Dan Shi, Guo-Qiu Dong, Ke-Ren Shen, Yao Pan, Shu-Mei Wei, Ying Chen, Ri-Sheng Yu

**Affiliations:** Department of Radiology (Shi D, Dong GQ, Shen KR, Pan Y, Chen Y, Yu RS), Department of Pathology (Wei SM), The Second Affiliated Hospital, Zhejiang University School of Medicine, Jiefang Road 88#, Hangzhou 310009, China.

**Keywords:** CT, MR, neuroendocrine tumor, retroperitoneum

## Abstract

**Rationale:**

Primary neuroendocrine tumors (NETs) of the retroperitoneum are extremely rare. The purpose of this case report is to highlight the unusual growth pattern and radiologic features of primary retroperitoneal NETs.

**Patient concerns:**

A 46-year-old woman was found to have a retroperitoneal cystic and solid mass during a physical checkup.

**Diagnoses:**

The mass was mainly multiseptated in the cystic portion and had a bead-like, lobulated appearance. The solid portion showed restricted diffusion on diffusion-weighted imaging and obvious homogeneous enhancement. The cystic portion showed ring-like and septal enhancement. The patient was diagnosed with a grade 2 (G2) NET of the retroperitoneum after surgery.

**Interventions:**

The patient underwent resection of the large retroperitoneal tumor.

**Outcomes:**

The patient returned 20 months later with tumor recurrence in the retroperitoneum. She was enrolled in a clinical trial for sulfatinib, and the mass was considerably reduced in size after 4 months. During a nearly 1.5-year follow-up, the mass gradually became slightly enlarged. The expression of somatostatin receptor 2 (SSTR2) was detected, and somatuline was administered as the current treatment.

**Lessons subsections:**

When a retroperitoneal mass presents as a well-defined cystic or solid hypervascular mass with a fibrous capsule, a primary retroperitoneal NET should be considered in the differential diagnosis.

## 1 Introduction

Neuroendocrine tumors (NETs) are heterogeneous in nature and have varying growth rates.^[1]^ Gastroenteropancreatic NETs (GEP-NETs) constitute the largest group of primary NETs that arise in the abdominal cavity. Small intestinal NETs are the most common NETs in the gastrointestinal tract, followed by pancreatic NETs.^[2]^ NETs found in the retroperitoneum are most often metastatic,^[3]^ and primary NETs of the retroperitoneum are extremely rare. We report a case of a middle-aged woman who was surgically confirmed to have a primary retroperitoneal NET, and the radiologic features of the NET were observed on computed tomography (CT) and magnetic resonance (MR) imaging. The purpose of this case report is to highlight the unusual growth patterns and radiologic features of primary retroperitoneal NETs. We also present a comprehensive review and summary of all reported cases in the pertinent English-language literature.

### 1.1 Consent

This retrospective case report was approved by the ethics committee of The Second Affiliated Hospital, Zhejiang University School of Medicine. Written informed consent was obtained from the patient for publication of this case report. A copy of the written consent is available for editorial review.

## 2 Case report

A 46-year-old woman was admitted to The Second Affiliated Hospital, Zhejiang University School of Medicine, due to the discovery of a lesion during a physical checkup at a local hospital. Ultrasonography and CT revealed a cystic and solid lesion in the retroperitoneum. She had no prior surgeries, and her medical history and family history could not explain the mass.

On admission, the results of the physical examination were unremarkable. Laboratory examination results were significant for hemoglobin (75 g/L) and D-dimer (1350 μg/L fibrinogen equivalent units (FEU)). Other laboratory tests, including those for tumor markers carbohydrate antigen 199 (CA199), carbohydrate antigen 125 (CA125), carcinoembryonic antigen (CEA), and alpha fetoprotein (AFP), were normal.

**Figure 1:**
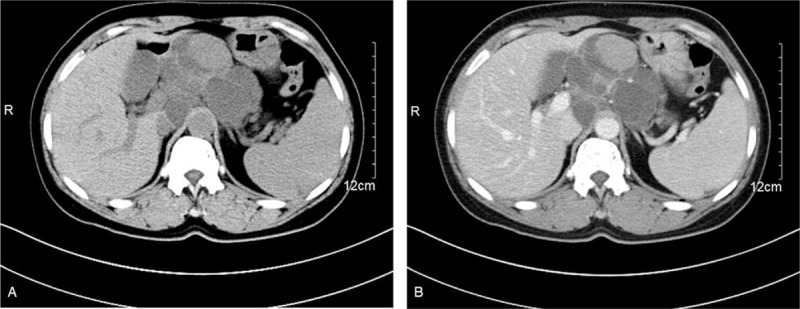
Abdominal CT images revealing a retroperitoneal cystic and solid mass located near the midline. (A) Axial nonenhanced CT image showing that the mass was mainly multiseptated in the cystic portion and had a bead-like, lobulated appearance. (B) Axial contrast-enhanced CT image showing a solid portion with obvious homogeneous enhancement from 42 HU to 73 HU and a cystic portion with ring-like and septal enhancement. CT = computed tomography, HU = Hounsfield units.

**Figure 2:**
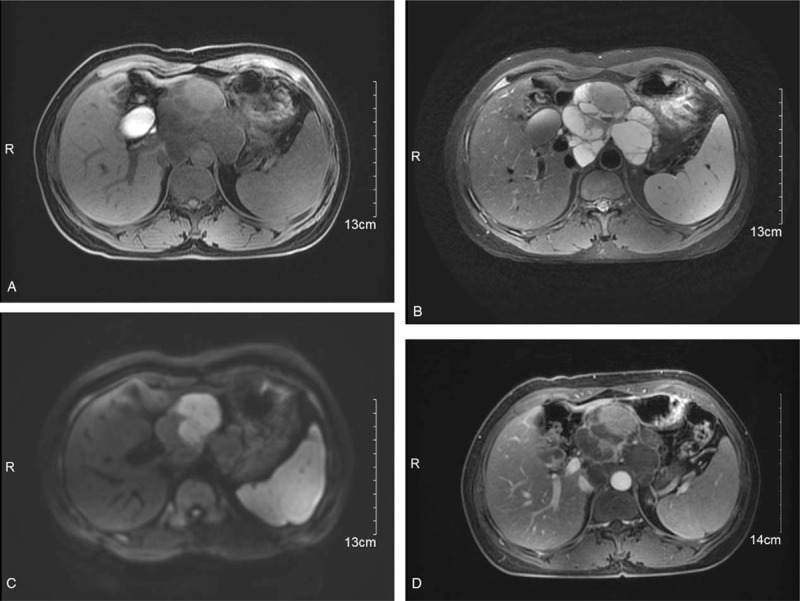
MR images showing a 9-cm × 8-cm cystic and solid mass in the retroperitoneum. (A) Axial fat-suppressed T1-weighted MR image showing a mass with heterogeneous hypointensity. (B) Axial T2-weighted MR image showing a mass with heterogeneous hyperintensity. (C) Diffusion-weighted image showing restricted diffusion in the solid portion. (D) Axial contrast-enhanced fat-saturated T1-weighted image showing marked and persistent enhancement of the solid portion and septations. MR = magnetic resonance.

After admission, abdominal CT and MR imaging were performed. Abdominal CT revealed a retroperitoneal cystic and solid mass located near the midline. The mass was adjacent to the lower left liver and partly wrapped around the pancreas. The mass was mainly multiseptated in the cystic portion and had a bead-like, lobulated appearance. The solid portion showed obvious homogeneous enhancement, with an increase from 42 Hounsfield units (HU) to 73 HU after contrast injection. The cystic portion showed ring-like and septal enhancement (Fig. 1A and B). MR imaging also showed a 9-cm × 8-cm cystic and solid mass in the retroperitoneum. The mass had heterogeneous hypointensity on T1-weighted imaging and heterogeneous hyperintensity on T2-weighted imaging. The solid portion showed restricted diffusion on diffusion-weighted imaging (Fig. 2A-D). The imaging diagnosis was a retroperitoneal tumor with a large area of cystic change, including plexus neurofibromatosis. Extensive evaluations did not reveal any evidence of primary tumors elsewhere in the body, and metastasis was not observed in the abdominal cavity.

Seven days after admission, the patient underwent resection of the large retroperitoneal tumor (June 7, 2016). An approximately 8-cm × 7-cm polycystic mass in the retroperitoneum was resected. During surgery, the retroperitoneal tumor was found to be located adjacent to the head of the pancreas, with a distinct border. The integrity of the pancreas was preserved. The solid portion of the mass was 3 cm in diameter and had moderate hardness. The mass wrapped around the left gastric artery, and the remainder had a well-defined margin. After removal of the tumor, the omentum, mesentery, diaphragm, retroperitoneum and pelvic cavity were explored by palpation, and no tumor-like masses were discovered.

**Figure 3:**
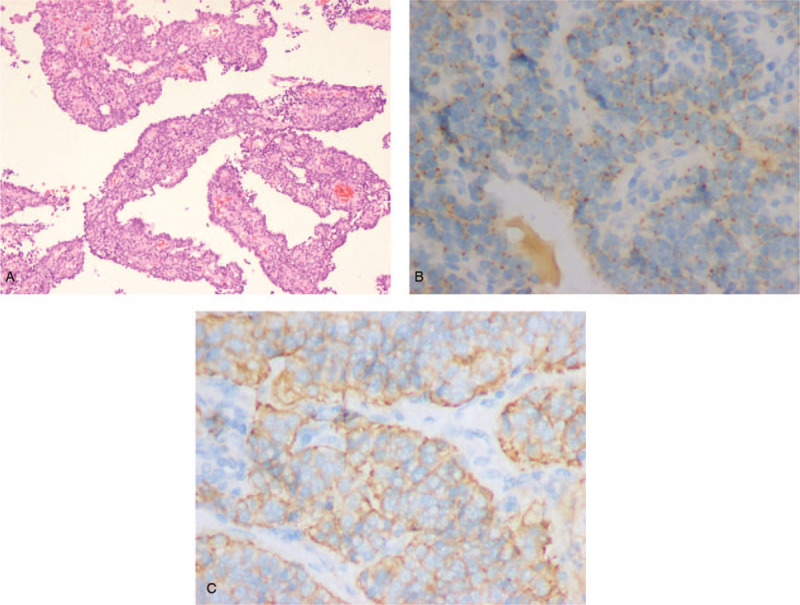
(A) Histopathology showing that the tumor was composed of oval and round epithelial cells with a papillary structure (hematoxylin-eosin staining, × 100). (B, C) Immunohistochemistry of the tumor cells (× 400). Tumor cells were positive for CgA (B) and Syn (C). CgA = chromogranin A, Syn = synaptophysin.

The resected mass was mainly cystic and solid. Cross-sections revealed that the mass had a “fish flesh” appearance and was soft. Microscopic examination revealed that the retroperitoneal mass was composed of oval and round epithelial cells with a papillary structure (Fig. 3A). Fewer than 2 mitoses were observed per 10 high-power fields (2/10 HPFs). Immunohistochemistry showed that the tumor cells were positive for CD56, chromogranin A (CgA), cytokeratin (CK) (AE1/AE3), CAM5.2, and synaptophysin (Syn) but negative for vimentin and progesterone receptor (PR) (Fig. 3B and C). The Ki-67 index reached approximately 5%. Based on the histopathological and immunohistochemical findings, the tumor was definitively diagnosed as a grade 2 (G2) NET of the retroperitoneum. The patient had an uneventful recovery and was discharged 1 week after surgery in good condition. Adjuvant endocrine therapy was rejected by the patient.

**Figure 4:**
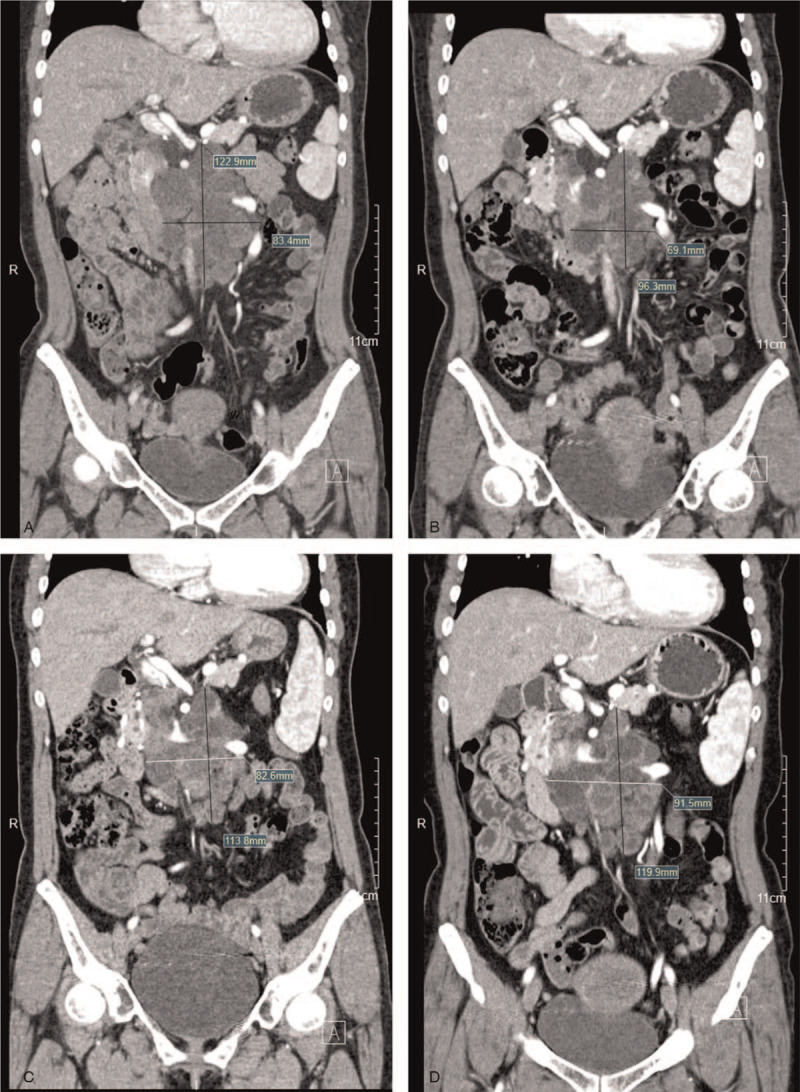
Coronal contrast-enhanced CT images at 4 follow-up examinations: January 24, 2018 (A); May 14, 2018 (B); April 22, 2019 (C); December 10, 2019 (D). **(**A-B**)** The mass was considerably reduced in size. (B-D) The mass gradually became slightly enlarged. CT = computed tomography.

The patient returned 20 months later with tumor recurrence in the retroperitoneum (Fig. 4A). She was enrolled in a clinical trial for sulfatinib (SANET-ep research), which was being evaluated for advanced nonpancreatic NETs. Every month, she took 300 mg of oral sulfatinib daily for 3 weeks and stopped for 1 week. The treatment response was evaluated according to the RESIST 1.1 criteria.^[4]^ The mass was considerably reduced in size after 4 months (Fig. 4B). During a regular follow-up examination nearly 1 year later, the mass was found to be slightly enlarged (Fig. 4C). At the subsequent 8-month follow-up (Fig. 4D), the mass was further slightly enlarged. The expression of somatostatin receptor 2 (SSTR2) was detected in postoperative specimens, and somatuline was administered as the current treatment. She was treated with 40 mg of intramuscular somatuline every 2 weeks.

## 3 Discussion

Retroperitoneal NETs have been found in the pancreas, duodenum, common bile duct and kidneys.^[5]^ However, primary nonmetastatic NETs arising from the retroperitoneum are extremely rare. In our case, the tumor did not arise from an alimentary organ, such as the pancreas or gut. On histologic examination, the tumor showed no signs of lymph node, paraganglia or pancreatic tissues within it. Moreover, an extensive evaluation did not reveal any evidence of primary tumors elsewhere in the body. Therefore, the mass in this case was likely a primary NET originating in the retroperitoneal cavity.

NETs constitute a group of tumors that originate from diffuse neuroendocrine cells throughout the body and are characterized by a wide spectrum of clinical manifestations. The classification proposed by the World Health Organization (WHO) was updated in 2010 to include the tumor grade and differentiation in the criteria. NETs are divided into well-differentiated, low-grade (G1, <2 mitoses/10 HPFs and a Ki-67 index <3%); well-differentiated, intermediate-grade (G2, 2–20 mitoses/10 HPFs or a Ki-67 index = 3%–20%); and poorly differentiated, high-grade (G3, >20 mitoses/10 HPFs and a Ki-67 index > 20%) lesions.^[6]^ The WHO proposed a new classification in 2017, and well-differentiated, high-grade (G3) NETs have now been officially defined as a subgroup of pancreatic high-grade (G3) NETs.^[7]^ Based on this classification, this patient was diagnosed with a well-differentiated, intermediate-grade (G2) NET.

We conducted a literature review of all the cases of primary retroperitoneal NETs published in the English-language literature to summarize the imaging features of retroperitoneal NETs^[5,8–13]^ (Table 1). To the best of our knowledge, the present case is the eighth to be reported and the second case to be reported in the Chinese population.

**Table 1:** Summary of primary retroperitoneal NETs.

Author, year	Age	Sex	Symptom	Location	Size	Grade	Gross Findings	Imaging Features
Yajima et al, (1984)^[8]^	41	Female	Lower abdominal pain	Retroperitoneal cavity, fixed in the base of the pelvis	5 × 7 × 8 cm	Endocrine Carcinoma	Homogenous and solid, grayish-yellow appearance	Solid with a smooth surface and thick fibrous capsule
Scapinello et al, (1995)^[9]^	71	Female	Left abdominal pain	Superolateral left retroperitoneum	21 cm	NET	Large cyst with a thin fibrous capsule, filled with hemorrhagic fluid	Large cyst with a thin fibrous capsule, filled with hemorrhagic fluid and contained a 5 cm parietal hemorrhagic nodule
Hong et al, (2012)^[10]^	37	Female	No (incidentally found)	Retroperitoneum, lateral side of the right kidney	/	NET	With a thick fibrotic capsule, filled with hemorrhagic fluid	With a thick fibrotic capsule, filled with hemorrhagic fluid
Yildrim et al, (2013)^[11]^	14	Male	Vomiting	Retroperitoneum, anterior side of the pancreas	3.5 × 3.5 cm	Well-differentiated (G1-G2) NET	Sharply demarcated solid grayish-tan colored lesion with punctuate foci of hemorrhage	Solid homogeneous mass
Dehal et al, (2015)^[12]^	65	Female	No (incidentally found)	Retroperitoneum, left of the midline adjacent to the left kidney, anterior side of left ureter	7.5 cm	Low-grade (G1), well-differentiated NET	Solid, ovoid lesion; a tan-red, focally hemorrhagic, and necrotic cut surface	Solid, ovoid, slightly inhomogeneous mass
Kwon, (2017)^[13]^	62	Female	Intermittent abdominal pain	Retroperitoneum, anterior side of the left kidney	9 cm	Intermediate-grade (G2), well-differentiated NET	Well-margined, thick-walled cystic lesion	Large, lobulated, round heterogeneous mass with peripheral thick capsule, mainly multiseptated cystic portion in the central area, peripheral rim enhancement
Ye et al, (2019)^[5]^	54	Male	Abdominal discomfort	Retroperitoneum, horizontal segment of the duodenum	3.5 × 4 × 3.5 cm	Low-grade (G1), well-differentiated NET	Pale and hard encapsulated with poor mobility	Circular, solid lesion, hyperattenuation on contrast-enhanced image
Shi et al, (2020) (This case)	46	Female	No (incidentally found)	Retroperitoneum, adjacent to the head of pancreas	8 × 7cm	Intermediate-grade (G2), well-differentiated NET	Cystic and the solid lesion, a “fish flesh” and soft appearance	Mainly cystic with multiseptation, bead-like, lobulated mass with homogeneous solid portion; restricted diffusion of solid portions on DWI; solid portions and septations showed marked and persistent homogeneous enhancement

NET = neuroendocrine tumor, G = grade, DWI = diffusion-weighted imaging.

According to the review of the published cases, retroperitoneal NETs occurred more commonly in the 4th-6th decades of life, with a median patient age of 54 years (range 14–71 years). A slight female predominance was noted. These NETs were asymptomatic, or the clinical symptoms were nonspecific (vomiting, abdominal pain and discomfort), and the symptoms resulted from the effect of the mass on adjacent structures. All patients had no manifestations of carcinoid syndrome, and no clinical evidence of hormone production (nonfunctioning tumors) was identified. Three patients (including our patient) had tumors that evolved asymptomatically and were incidentally diagnosed on an abdominal imaging examination.

Most retroperitoneal NETs were relatively large in size, with a median size of 8 cm (range 4–21 cm) at the time of detection. The retroperitoneum provides a large space for tumors to grow; thus, the tumors were not detected until they were very large. Only 3 patients (Dehal et al,^[12]^ Kwon,^[13]^ and Ye et al)^[5]^ had tumors that were detectable on CT, and our patient underwent both CT and MR imaging. On imaging, the retroperitoneal NETs typically appeared as well-defined, hypervascular masses due to their rich capillary networks. Small tumors tended to be round or ovoid homogeneous solid masses. Large tumors were commonly lobulated and heterogeneous with cystic, hemorrhagic, and necrotic areas. Calcification was not observed. Most cases had a fibrous capsule and showed enhanced capsules in post-enhancement images from delayed phase imaging. These tumors were mainly cystic and typically had a hypervascular rim. The enhancement of the tumors was homogeneous, ring-like, or heterogeneous. In our patient, the mass was mainly multiseptated in the cystic portion and had an irregular shape with a bead-like, lobulated appearance, which was quite different from the other cases. Retroperitoneal NETs were classified as G1-G2. The Ki-67 and mitotic indexes were not provided in some early cases; thus, those cases could not be accurately classified. No cases were associated with lymphatic, hepatic, or other metastases. The retroperitoneal NETs were sporadic, and no cases were associated with any familial syndromes.

The differential diagnosis of a retroperitoneal cystic and solid mass includes plexiform neurofibroma, lymphangiomyomatosis (LAM) and primary retroperitoneal mucinous cystadenocarcinoma (PRMC). Plexiform neurofibromas are almost exclusively observed in neurofibromatosis type 1 (NF l). These masses are typically bilateral, symmetric, low-attenuation masses, and the lumbosacral plexus is the most common site in the retroperitoneum.^[14]^ LAM is a rare systemic disorder that occurs almost exclusively in women of childbearing age. In patients with extrapulmonary LAM (E-LAM), mediastinal and upper abdominal retroperitoneal lymphadenopathies (LAPs), and renal angiomyolipomas are common.^[15]^ A lymphangioleiomyoma is a cystic mass found in the lymphatic system. The signal intensities of the center area on T1-weighted imaging and T2-weighted imaging are homogeneous and similar to those of ascites. The peripheral region is immediately enhanced, and homogeneous delayed enhancement of the entire mass is observed.^[16]^ PRMC is an extremely rare neoplasm with a female predilection that is found almost exclusively in the lateral retroperitoneal spaces. This tumor is a retroperitoneal cystic lesion with solid mural nodules that show progressive enhancement on enhanced CT.^[17]^ Our patient was a 46-year-old woman without any familial syndromes. The tumor contained a solid portion and was mainly multiseptated in the cystic portion, with a bead-like, lobulated appearance. The tumor was located in the middle-upper central retroperitoneum. Therefore, the 3 aforementioned differential diagnoses were not supported.

Surgical resection is the first-line treatment for primary NETs and is potentially curative, even in cases of metastatic disease, regardless of the NET origin if at least 90% of the tumor can be successfully removed.^[18–20]^ The previous case reports indicated that most retroperitoneal NETs grow slowly and have a limited risk for local invasion and metastasis.^[5]^ In this case, metastasis was not observed, and the patient was treated with surgical resection without postoperative adjunctive therapy. This patient experienced recurrence in the retroperitoneum 20 months later based on CT scans, and the mass gradually became enlarged with sulfatinib treatment. SSTR2 expession was detected in this patient, and somatuline (somatostatin analogue, SSA) was used as a palliative treatment option. Recent research has demonstrated that SSAs exert antiproliferative effects and inhibit tumor growth by binding the SSTR2. SSA treatment may prolong both overall and progression-free survival in patients with NETs, and multiple trials have demonstrated high rates of disease stabilization upon treatment with SSAs.^[21]^ However, this patient showed recurrence in the retroperitoneum, which was not observed in previous cases, indicating that retroperitoneal NETs may have a risk for recurrence and metastasis. The prognosis of retroperitoneal NETs is relatively good, but the role of postoperative adjunctive therapy and the therapeutic options for recurrent NETs are still undetermined.

## 4 Conclusion

We present an extremely rare case of a primary NET that arose from the retroperitoneum. When a retroperitoneal mass presents as a well-defined cystic and/or solid hypervascular mass with a fibrous capsule, a primary retroperitoneal NET should be considered in the differential diagnosis.

## Acknowledgment

We thank Xin-Feng Yu for his excellent help in the preparation of the manuscript.

## Author contributions

**Conceptualization**: Dan Shi, Guo-Qiu Dong, Ri-Sheng Yu.

**Data curation**: Dan Shi, Guo-Qiu Dong, Ke-Ren Shen.

**Formal analysis**: Ke-Ren Shen, Yao Pan, Shu-Mei Wei.

**Methodology**: Dan Shi, Ying Chen, Ri-Sheng Yu.

**Resources**: Dan Shi, Guo-Qiu Dong, Ke-Ren Shen, Yao Pan, Shu-Mei Wei, Ying Chen, Ri-Sheng Yu.

**Supervision**: Ying Chen, Ri-Sheng Yu.

**Writing – original draft**: Dan Shi.

**Writing – review & editing**: Shu-Mei Wei, Ri-Sheng Yu.

## Abbreviations

Abbreviations: CT = computed tomography, GEP-NET = gastroenteropancreatic neuroendocrine tumor, LAM = lymphangiomyomatosis, MR = magnetic resonance, NET = neuroendocrine tumor, PRMC = primary retroperitoneal mucinous cystadenocarcinoma, SSA = somatstatin analogue, SSTR2 = somatostatin receptor 2.
